# Effects of Silicon Content on the Microstructures and Mechanical Properties of (AlCrTiZrV)-Si_x_-N High-Entropy Alloy Films

**DOI:** 10.3390/e21010075

**Published:** 2019-01-16

**Authors:** Jingrui Niu, Wei Li, Ping Liu, Ke Zhang, Fengcang Ma, Xiaohong Chen, Rui Feng, Peter K. Liaw

**Affiliations:** 1 School of Materials Science and Engineering, University of Shanghai for Science and Technology, Shanghai 200093, China; 2 Department of Materials Science and Engineering, The University of Tennessee, Knoxville, TN 37996, USA

**Keywords:** (AlCrTiZrV)-Si_x_-N films, microstructure, mechanical property, nanocomposite structure

## Abstract

A series of (AlCrTiZrV)-Si_x_-N films with different silicon contents were deposited on monocrystalline silicon substrates by direct-current (DC) magnetron sputtering. The films were characterized by the X-ray diffractometry (XRD), scanning electron microscopy (SEM), high-resolution transmission electron microscopy (HRTEM), and nano-indentation techniques. The effects of the silicon content on the microstructures and mechanical properties of the films were investigated. The experimental results show that the (AlCrTiZrV)N films grow in columnar grains and present a (200) preferential growth orientation. The addition of the silicon element leads to the disappearance of the (200) peak, and the grain refinement of the (AlCrTiZrV)-Si_x_-N films. Meanwhile, the reticular amorphous phase is formed, thus developing the nanocomposite structure with the nanocrystalline structures encapsulated by the amorphous phase. With the increase of the silicon content, the mechanical properties first increase and then decrease. The maximal hardness and modulus of the film reach 34.3 GPa and 301.5 GPa, respectively, with the silicon content (x) of 8% (volume percent). The strengthening effect of the (AlCrTiZrV)-Si_x_-N film can be mainly attributed to the formation of the nanocomposite structure.

## 1. Introduction

Hard coating tools are the fastest developing new techniques and have become the symbol of modern cutting tools. Among them, the most widely-used coatings are binary and ternary systems of traditional metal nitrides or oxides coatings, such as titanium nitrides (TiN), chromium nitrides (CrN), etc. [1]. With the development of the coating science and technology, nanocomposite films with better mechanical properties have become a research hotspot due to their higher mechanical properties, thermal stability, and oxidation resistance. In 1992, Li et al. [2] first prepared TiSiN nanocomposite films with a hardness of over 60 GPa using the physical vapor deposition (PVD), and suggested that the films consisted of TiN and Si_3_N_4_ phases, in which TiN was crystalline, while Si_3_N_4_ was amorphous. In 2000, Veprek et al. [3] prepared the TiSiN nanocomposite films with the superhigh hardness of 80–105 GPa, which aroused great interest among researchers all over the world. Since then, researchers have done a great amount of research on the preparation of nanocomposite films and their hardening mechanism, and have proposed the widely-accepted model of nanocrystals encapsulated by the amorphous interfacial phase, which is named as the *nc*-TiN/*a*-Si_3_N_4_ model (*nc* refers to nanocrystals, and *a* refers to amorphous) [4]. 

In 2004, Yeh et al. innovatively proposed the concept of high-entropy alloys (HEAs), and Cantor established the equiatomic multicomponent alloys [5,6]. HEAs are a high-entropy solid-solution phase alloy formed by five or more principal elements, each of which has an atomic concentration between 5–35 at.% (atomic percent). The HEAs are not inclined to form intermetallic compounds due to their high-entropy effect, which tend to stabilize the simple body-centered-cubic (BCC), face-centered-cubic (FCC), or hexagonal-close-packed (HCP) solid solution [7,8,9,10,11,12]. A large number of subsequent studies have shown that HEAs have many superior properties than conventional alloys, such as the high strength, great hardness, strong wear resistance, good fatigue resistance, high oxidation and corrosion resistance [13,14,15,16,17,18,19,20,21,22,23,24,25,26,27,28]. With the development of new HEAs, studies of HEA nitride films have also attracted the great interest of many researchers. Feng et al. [29] deposited the (ZrTaNbTiW)N films on the substrate of the Ti_6_Al_4_V alloy by multi-target magnetron sputtering to investigate the composition, structure, and mechanical properties of the film. They found that the (ZrTaNbTiW)N film showed the BCC and FCC structures, and its mechanical properties were significantly greater than those of ZrTaNbTiW films. Cheng et al. [30] deposited multi-component (AlCrTaTiZr)-Si_x_-N films on the monocrystalline Si substrate by reactive radio-frequency (RF) magnetron sputtering and probed the influence of the silicon content on the structures, morphologies, and mechanical properties of the films. The results showed that the incorporation of silicon significantly increased the oxidation resistance of AlCrTaTiZr nitride films, but lowered their hardnesses. Tsai et al. [12] deposited the multi-component (AlCrMoTaTi)N films with different silicon contents by reactive RF magnetron sputtering and studied the effects of silicon contents on the nitride films. Their results showed that the incorporation of silicon led to the lattice distortion of the films, improved the mechanical properties of the films, but reduced the electrical properties of the films. Moreover, Tsai et al. [31] deposited the (AlCrMoTaTi)-Si_x_-N films on the Si substrate by the reactive RF magnetron sputtering to systematically study the effects of the silicon content on the oxidation of the films. It was found that the oxidation resistance of the films improved with the increase of the silicon content, which could be attributed to the existence of Al and Si in the films.

Based on the current research situation, it can be seen that there is no clear conclusion about the effects of the Si element on the microstructures and mechanical properties of HEA nitride films. Therefore, this study combines the concepts of the “nanocomposite film” and “HEA nitride films” to prepare (AlCrTiZrV)-Si_x_-N films with different silicon contents by the reactive direct-current (DC) magnetron sputtering. The effects of silicon contents on the microstructures and mechanical properties of the films were systematically studied with the expectation of providing the experimental and theoretical basis for the application of the nanocomposite HEA nitride film in the industrial field.

## 2. Experimental 

### 2.1. Film Preparation

**Figure 1:**
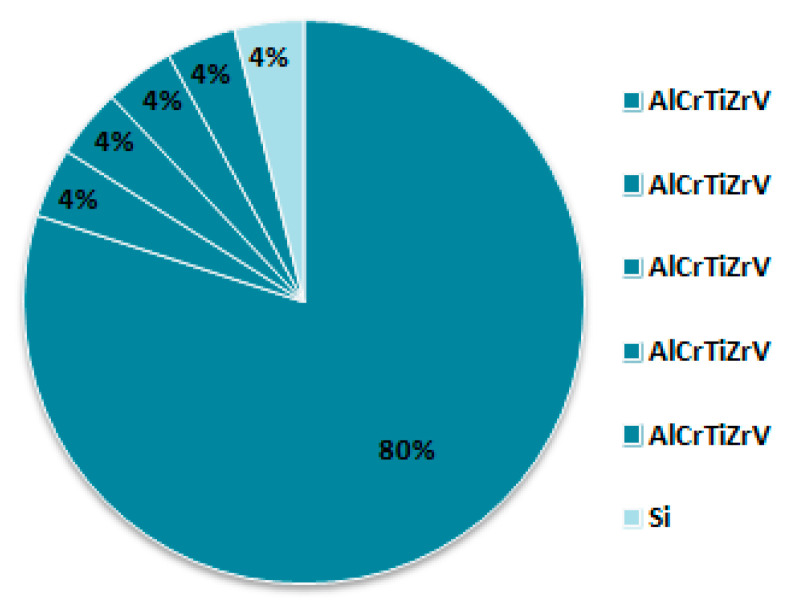
Schematic illustration of the AlCrTiZrVSi_x_ composite target.

The (AlCrTiZrV)-Si_x_-N films with different silicon contents were deposited on monocrystalline Si (100) wafers with a size of 35 mm × 25 mm × 0.5 mm using a JPG–450 multi-target magnetron sputtering system. The wafers were ultrasonically cleaned with the acetone and absolute ethanol for 15 min, and then dried into the sputtering chamber. The target used for sputtering is a self-made compound target with the structure shown in Figure 1. The process of preparing the compound target can be summarized as follows. Both the Si target and the equal-moles AlCrTiZrV target, with the diameter of 75 mm and the purity of 99.99% (volume percent), were cut down 5 slices of 25 equal fan segments by a low speed wire electrical discharge machine (EDM). Then, keeping the 80% (volume percent) of the AlCrTiZrV target unchanged, these fan segments were assembled into the composite target with different silicon contents by changing the number of the Si sector. For example, the composite target shown in Figure 1 has only one Si sector, therefore the Si content (x) of the composite target is 4% (volume percent). According to this method, composite targets with the Si contents (x) of 4%, 8%, 12%, and 16% can be assembled for sputtering.

The AlCrTiZrVSi_x_ compound target was controlled by a DC power supply. The base pressure was pumped down to 3.0 × 10^−4^ Pa before the deposition. During the experiment, the Ar and N_2_ flow rates were both maintained at the 10 standard cubic centimeter per minute (sccm). The working pressure was adjusted to 0.7 Pa, and the sputtering power was 180 W. To improve the homogeneity of films, the substrate was rotated at a speed of 10 r/min. The deposition time was 1.5 h, and finally the (AlCrTiZrV)-Si_x_-N films with a thickness of about 1.8 μm were obtained.

### 2.2. Film Characterization and Measurement

A series of characterization and testing of the deposited (AlCrTiZrV)-Si_x_-N films were performed. The structural and phase analyses were conducted on the D8 Advance X-ray diffractometer (XRD, Bruker, Germany) using the CuK_α_ radiation (λ = 0.15406 nm) with a measurement range of 25° to 90° and the Jade software, respectively. The microstructures of the films were observed by the Quanta FEG450 field emission environmental electron microscope (SEM, FEI, USA) and Tecnai G^2^20 high-resolution field-emission transmission electron microscope (HRTEM, FEI, USA). The NANO Instrument, a G200 nano indenter (Agilent, USA) with the Berkovich indenter was used to study the mechanical properties. The loading and unloading curves were obtained by accurately recording the change of the loading depth with the load. Then the hardness and elastic modulus were calculated by the Oliver–Pharr model [32]. During the measurement, the loading depth was set to 100 nm, less than 1/10 of the thickness of the films, so as to eliminate the effect of the substrate on the measurements. Each hardness or elastic modulus value was an average of at least 16 measurements.

## 3. Results

**Figure 2:**
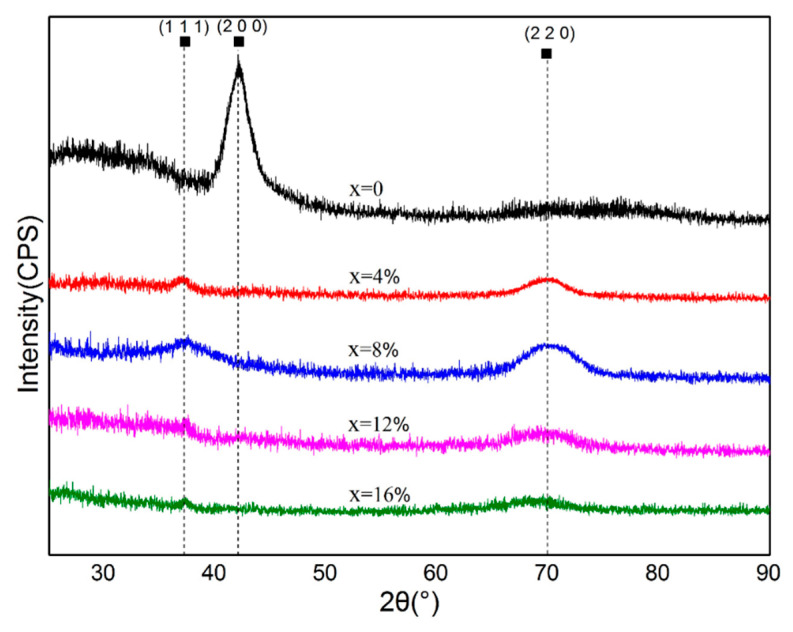
X-ray diffraction (XRD) patterns of (AlCrTiZrV)-Si_x_-N films with different silicon contents.

The XRD patterns of the (AlCrTiZrV)-Si_x_-N films with different silicon contents are presented in Figure 2. It can be seen that the film without the Si element shows a simple FCC structure with a diffraction peak near 2θ = 41°, corresponding to the reflection of the (200) crystal plane, suggesting that an FCC-structured solid-solution phase is formed with good crystallinity, and no other complicated intermetallic compound is developed in the films. The reason for this phenomenon is that, due to the high-entropy effect, the HEA tends to form the stable solid-solution phase rather than intermetallic compounds. In bulk alloys of similar compositions to AlCrTiZrV, several researchers have reported the formation of an order B2 structure [33,34], while in this investigation, the crystal phase of the film without Si is a disordered FCC structure. This phenomenon may contribute to the reaction of the HEA with N_2_. The films form the HEA nitride rather than the simple HEA, which leads to the change of the structure of the crystal phase. Combined with the phase analysis of the Jade software, it can be determined that the solid-solution phase is composed of five binary nitrides from Al, Cr, Ti, Zr, and V. This result has been reported in previous works and proven to be thermodynamically stable due to the high mixing entropy effect [35,36].

With the incorporation of Si, no obvious diffraction peak can be observed at 2θ = 41°, indicating that the (200) diffraction peaks of films disappear with the incorporation of Si. From the XRD patterns of (AlCrTiZrV)-Si_x_-N (x = 4%, 8%, 12%, and 16%) film, it can be seen that the diffraction patterns have two diffraction peaks with low intensities at about 2θ = 37° and 2θ = 70°, corresponding to fcc (111) and (200) peaks, respectively, suggesting that the film crystallinity decreases with the incorporation of the Si element, relative to the (AlCrTiZrV)N film, and the microstructure may be nanocrystalline or amorphous.

**Figure 3:**
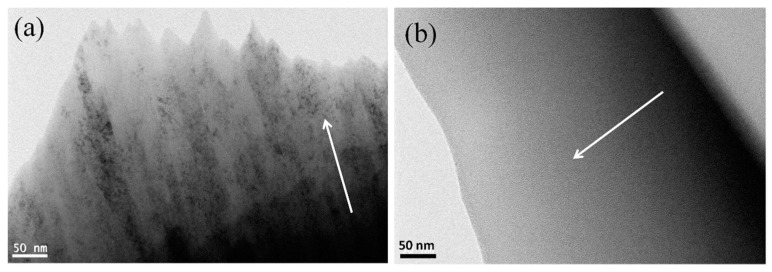
Low-magnification cross-sectional transmission electron microscope (TEM) images of (AlCrTiZrV)-Si_x_-N films: (**a**) (AlCrTiZrV)N; (**b**) (AlCrTiZrV)-Si_0.08_-N.

Figure 3a shows that the film without the Si element grows in a columnar crystal with the growth direction marked by the arrow. Figure 3b suggests that the (AlCrTiZrV)-Si_0.08_-N film exhibits the amorphous morphology with the growth direction indicated by the arrow. In addition, with the incorporation of Si, the diffraction peaks of the films move to high angles, as presented in Figure 2, suggesting that the lattice parameters of the nitride phase decrease, which may be attributed to the fact that the doped Si atoms can be dissolved into the (AlCrTiZrV)N lattice and occupy some lattice positions. As the radius of the Si atom is smaller than that of other metal elements, the lattice parameters of the nitride phase decrease, which has been confirmed in other studies of ternary Si-contained nitride films [37,38]. Besides, it is clear to see the change of the lattice parameters of the nitride phase according to the values given by Table 1. 

**Figure 4:**
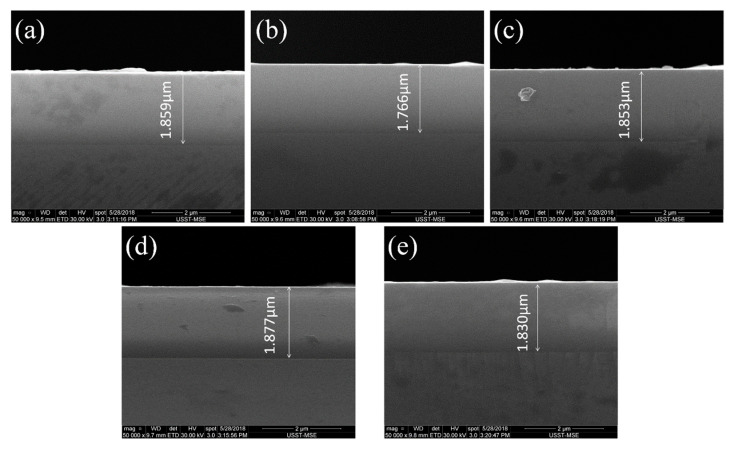
Cross-sectional SEM images of the (AlCrTiZrV)-Si_x_-N films. (**a**) (AlCrTiZrV)N; (**b**) (AlCrTiZrV)-Si_0.04_-N; (**c**) (AlCrTiZrV)-Si_0.08_-N; (**d**) (AlCrTiZrV)-Si_0.12_-N; (**e**) (AlCrTiZrV)-Si_0.16_-N.

The cross-sectional SEM images of the (AlCrTiZrV)-Si_x_-N films with different silicon contents are presented in Figure 4. It can be seen that the nitride films are well bonded to the substrates, and there is no obvious micro-gap between them. Moreover, the nitride films are grown uniformly with thickness of about 1.8 μm, and the surface and internal quality of the nitride films are good. All the films have the small surface roughness and a very dense and smooth cross-sectional structure without the visible grain feature. Table 2 shows the calculated grain-sizes values of the (AlCrTiZrV)-Si_x_-N films with different silicon contents according to the Scherrer’s equation. This trend indicates that the grain sizes of the films reach the nanometer level.

**Figure 5:**
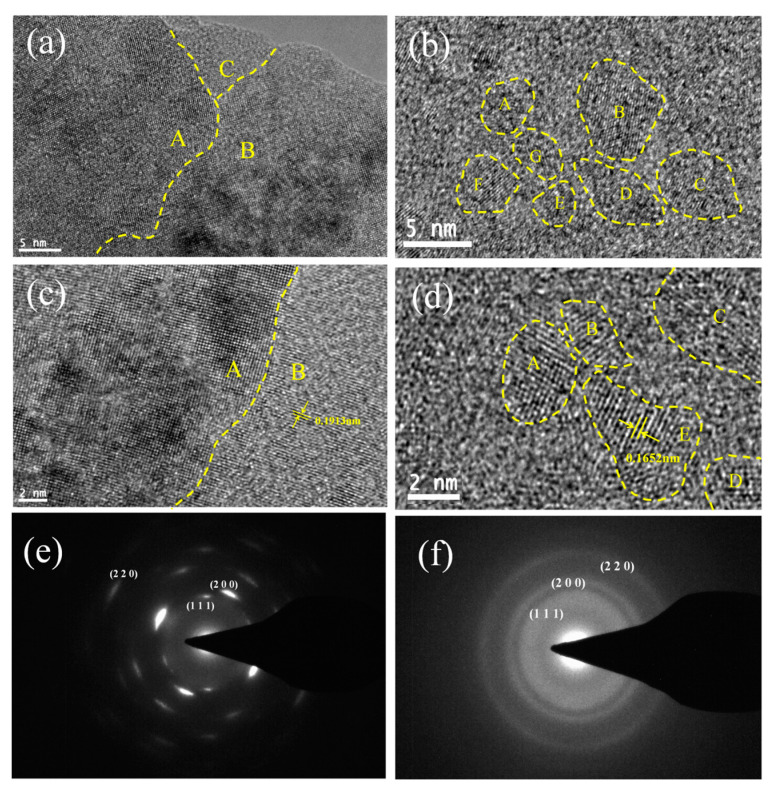
Cross-sectional HRTEM images and selected-area electron diffraction (SAED) patterns of the (**a**,**c**,**e**) (AlCrTiZrV)N and (**b**,**d**,**f**) (AlCrTiZrV)-Si_0.08_-N films: (**a**,**b**) low-magnification HRTEM images; (**c**,**d**) high-magnification HRTEM images; (**e**,**f**) SAED patterns.

The cross-sectional HRTEM images of the (AlCrTiZrV)N and (AlCrTiZrV)-Si_0.08_-N films are presented in Figure 5. Figure 5a,c is the microstructures of the (AlCrTiZrV)N film, in which A, B, and C represent different crystal grains. Figure 5b,d shows the images of the (AlCrTiZrV)-Si_0.08_-N film, where A, B, C, D, E, F, and G denote the nanocrystals inside the films. Figure 5e,f presents the selected-area electron diffraction (SAED) patterns of two films. A comparison of Figure 5a,b indicates that the grains of the (AlCrTiZrV)N film (the areas marked by A, B, and C) are comparatively coarse, reaching tens or hundreds of nanometers, while the grains of the (AlCrTiZrV)-Si_0.08_-N film (inside the yellow dotted line) are much smaller, approximately 2–5 nm, suggesting that the incorporation of the Si element can effectively refine the grain of the film to the nanometer level, which also confirms the previous XRD result in Figure 2. According to Figure 5c,d, it can be seen that there is no other phase existing between the grains of the (AlCrTiZrV)N film (on both sides of the yellow dotted line) from the high-magnification HRTEM, and the grains’ contact directly with each other. While the grains of the (AlCrTiZrV)-Si_0.08_-N film do not contact directly, and there are some reticular amorphous interfacial phases between two grains, that is, between two yellow closed dotted lines. It is speculated that the interfacial phase is the amorphous phase and corresponds to the amorphous projection of the XRD pattern. For the SAED patterns images, the FCC structure with the (200) preferred orientation of the (AlCrTiZrV)N film could be seen in Figure 5e, while the SAED patterns of the (AlCrTiZrV)-Si_0.08_-N film in Figure 5f exhibit the nanocrystalline and amorphous features, which are consistent with the XRD analysis of Figure 2.

**Figure 6:**
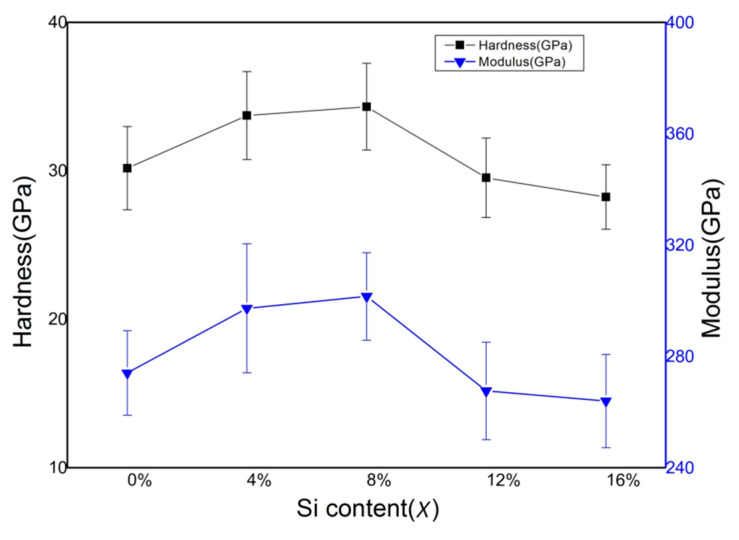
Effect of silicon content on mechanical properties of (AlCrTiZrV)-Si_x_-N films.

The effects of Si contents on the mechanical properties of the (AlCrTiZrV)-Si_x_-N films are presented in Figure 6. The hardness and elastic modulus of the (AlCrTiZrV)N film are 30.1 GPa and 274.0 GPa, respectively. With the incorporation of Si, the hardness and elastic modulus of the films both first increase and then decrease. When the Si content (x) is 8%, the hardness and elastic modulus of the film reach the maximum, which are 34.3 GPa and 301.5 GPa, respectively. As the Si content (x) further increases to 12% or 16%, the hardness and elastic modulus of the films decrease and are lower than those of the (AlCrTiZrV)N film. It can be seen that the incorporation of Si has a significant effect on the mechanical properties of the films, which can be attributed to the microstructure change resulting from the incorporation of the Si element.

## 4. Discussion

### 4.1. The Formation of the Nanocomposite Structures

The nanocomposite film consisting of two insoluble phases presents a three-dimensional network structure film in which the interfacial phase encapsulates the matrix phase. The difficulty for the formation of the nanocomposite structure within the HEA nitride films lies in the remarkable mixing entropy effect, which enhances the mutual solubility of constituent elements. As a result, the Si atoms may be inclined to be incorporated in the crystalline lattice of the HEA nitride, rather than be segregated as the SiN_x_ phase, which can serve as the interfacial phase of the nanocomposite structure.

For example, Tsai et al. [30] investigated the effects of the silicon content (0–7.51 at.%) on the (AlCrMoTaTi)N coatings by the reactive-magnetron sputtering, and no formation of the nanocomposite structure had been reported. In particular, the formation of the SiN_x_ phase was not observed even after the Si content was increased to 7.51 at.%. They attributed the extended solubility of Si to the high entropy effect. Lin et al. [39] reported the formation of the nanocomposite structure in the (AlCrTaTiZr)SiN coatings produced by the reactive RF-magnetron sputtering, and found that the nanocrystalline phase is elongated, rather than equiaxed, which is a typical feature of the nanocomposite films. Moreover, the lattice can be observed within the denoted “amorphous regions”, suggesting the probable crystallized feature in these regions. More importantly, the composition distribution had not been provided in these investigations, making it difficult to verify the phase segregation within the coatings. 

**Figure 7:**
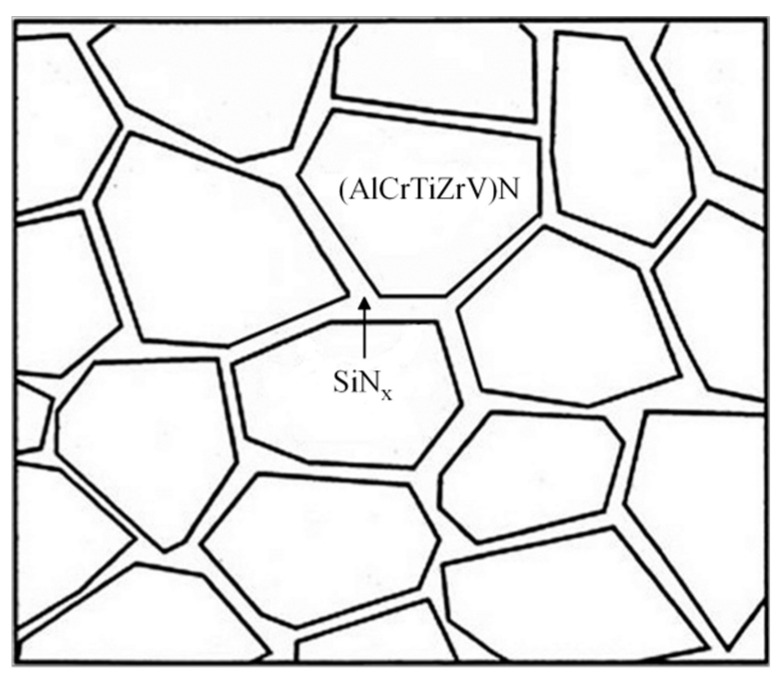
Schematic diagram of the nanocomposite structure of the (AlCrTiZrV)-Si_x_-N films.

Compared with the above results, in this investigation, through the high-magnification HRTEM image shown in Figure 5d, the equiaxed regions (the areas marked by A, B, C, D, and E) exhibit an ordered lattice structure, which is the HEA nitride phase, while the areas between two yellow closed dotted lines present an amorphous structure, which is the interfacial phase. Meanwhile, no lattice structure is observed in the amorphous region, indicating that the nanocomposite structure forms within the film resulting from the phase separation between the HEA nitride matrix and SiN_x_ interfacial phase. Moreover, according to the studies of Prochazka [40], the *a*-Si_3_N_4_ phase has the limited solubility in FCC nitrides, and the thermodynamically-driven phase separation will occur during the deposition, which may be the possible reason why the nanocomposite structures could form in the (AlCrTiZrV)-Si_x_-N films. Figure 7 is the schematic diagram of nanocomposite structures of the (AlCrTiZrV)-Si_x_-N films, in which the (AlCrTiZrV)N matrix phase is a nano-equiaxed crystal, and the SiN_x_ interfacial phase is a network amorphous phase.

### 4.2. The Strengthening Mechanism of the (AlCrTiZrV)-Si_x_-N Films

The mechanical properties of the films are improved with the proper addition of the Si element, which can be attributed to the microstructure change due to the Si incorporation. The reasons for improving their mechanical properties can be specifically discussed as follows.

According to the theory of solid-solution hardening [41], the incorporation of the Si element can improve the mechanical properties by causing the lattice distortion of the nitride films. To some extent, Table 1 shows that the incorporation of the Si element causes the lattice distortion of the nitride films. However, compared with the nitride film without Si, the mechanical properties of the nitride films with the high Si content are lower, indicating that the solid-solution strengthening is not the major factor in changing the mechanical properties of the nitride films.

From the classical Hall–Petch relationship [42]:

H = H_0_ + k_HP_d^−1/2^, (1)

where H_0_ is the intrinsic hardness of the (AlCrTiZrV)N, d is the average grain size, and k_HP_ is the Hall-Petch coefficient of (AlCrTiZrV)N. The reduction of the grain size could lead to the strengthening effect of the HEA-nitride film. According to the HRTEM analysis, it can be seen that with the incorporation of the Si element, the grain sizes of the nitride films significantly decreases to the nanometer scale. Besides, Table 2 shows that the grain sizes of the (AlCrTiZrV)-Si_x_-N films reduce with the incorporation of the Si element. Therefore, the effect of the fine-grained strengthening improves the mechanical properties of the nitride films to some extent. However, from the previous analysis, the mechanical properties of the nitride films with the low Si content increase, while the properties with the high Si content further decrease, that is, when the grain sizes reach a minimum, the mechanical properties of the nitride films are worst, suggesting that the fine-grained strengthening mechanism is not the main factor in strengthening the film.

From the HRTEM images and relevant analysis, the nanocomposite structure forms within the HEA nitride films. Combining the strengthening mechanism of the nanocomposite film (*nc*-TiN/*a*-Si_3_N_4_ model) [4,43,44], with the incorporation of Si, there are many equiaxed crystals with the nanometer size formed in the films, leading to the fact that the generation or multiplication of dislocations cannot happen within the films. At the same time, the thickness of the amorphous interfacial phases formed between the nanocrystals is small, and the crack is difficult to expand in the interfacial phases. Hence, the nitride films present a superhardness effect, and the mechanical properties are improved. Moreover, the quantity of the interfacial phases increases with the increase of the Si content, which makes the grains more refined (as shown in Table 2) and, therefore, effectively strengthens the film. Patscheider et al. [44] pointed out that the hardness of nanocomposite films peaks at the common minimum of the grain size of the crystalline phase and the grain separation by the amorphous phase. Namely, two conditions have to be fulfilled to achieve the maximum hardness for the nanocomposite film: the nanocrystals are approximately 5 nm in size and the mean distance has to be very small, about a few nanometers. Therefore, as shown in Table 2 and Figure 5d, when the average grain size of the crystalline phase is 4.36 nm, and the thickness of the amorphous interfacial phase is approximately 0.5–2 nm, the mechanical properties of the nitride films are the best.

However, if the interfacial phase thickness is too large, the hardness is mainly governed by the properties of the amorphous interfacial phase [44]. In this investigation, when the Si content is 8%, the thickness of the amorphous interfacial phase in the film could reach 2 nm. Because of the phase separation driven by thermodynamics, the (AlCrTiZrV)N matrix phase and the SiN_x_ interfacial phase are incompatible with each other. The Si only exists in the amorphous region. Thus, when the Si content is above 8%, the thickness of the amorphous interfacial phase will be 3–4 nm or even thicker. While at this time, the grain size of the crystalline phase is only 2–3 nm or even smaller, the hardness of the nitride film is mainly governed by the properties of the amorphous interfacial phase. This trend means that the hardness of the film approaches that of SiN_x_. The SiN_x_ phase is softer than the (AlCrTiZrV)N phase; meanwhile, the crack can expand within the amorphous interfacial phase due to the thickening of the interface, thus leading to the decrease of the mechanical properties. Hence, the mechanical properties of the films with the high Si content are lower than those of the film without Si. Therefore, it is believed that the nanocomposite structure is the main reason for the improvement of the mechanical properties of the HEA nitride films.

Due to the limitation of characterization techniques, however, it is difficult to accurately predict the strengthening effect from the nanocomposite structure in this investigation. As a result, more research should be carried out on the phase-segregation behavior of the Si-containing HEA nitride films to help investigate the strengthening behavior of nanocomposite structures by the advanced techniques, such as atom probe tomography. The relevant modeling and simulations are also needed.

## 5. Conclusions

(i) The (AlCrTiZrV)N film is a solid-solution phase with the FCC structure and has a (200) preferential orientation. With the incorporation of the Si element, the (200) diffraction peak of (AlCrTiZrV)-Si_x_-N films disappeared.

(ii) The (AlCrTiZrV)N film grows with a columnar crystal-growth mode. After the Si element is incorporated, the (AlCrTiZrV)-Si_x_-N films exhibit an amorphous fracture morphology. Moreover, the incorporation of the Si element effectively refines the grains of the nitride films, forming nanocrystals with a size of about 2–5 nm, and a large number of amorphous interfacial phases form simultaneously.

(iii) The nanocomposite structure is formed within the (AlCrTiZrV)-Si_x_-N films with the incorporation of the Si element, and the mechanical properties are improved. When the Si content (x) is 8%, the hardness and elastic modulus of the (AlCrTiZrV)-Si_x_-N films reach 34.3 GPa and 301.5 GPa, respectively. However, the further increase of the Si content will result in the deterioration of the mechanical properties of the (AlCrTiZrV)-Si_x_-N films.

## Figures and Tables

**Table 1:** The values of the lattice parameters of the (AlCrTiZrV)-Si_x_-N films. (d_1_ are the calculated values according to the XRD; d_2_ are the measurements according to the high-resolution transmission electron microscope (HRTEM)).

(AlCrTiZrV)-Si_x_-N	d_1_(nm)	d_2_(nm)
(AlCrTiZrV)N	0.2018	0.1913
(AlCrTiZrV)-Si_0.04_-N	0.1631	-
(AlCrTiZrV)-Si_0.08_-N	0.1629	0.1652
(AlCrTiZrV)-Si_0.12_-N	0.1627	-
(AlCrTiZrV)-Si_0.16_-N	0.1624	-

**Table 2:** The calculated grain-sizes values of the (AlCrTiZrV)-Si_x_-N films.

(AlCrTiZrV)-Si_x_-N	D(200)	D(220)
(AlCrTiZrV)N	22.18 nm	-
(AlCrTiZrV)-Si_0.04_-N	-	5.32 nm
(AlCrTiZrV)-Si_0.08_-N	-	4.36 nm
(AlCrTiZrV)-Si_0.12_-N	-	3.20 nm
(AlCrTiZrV)-Si_0.16_-N	-	2.60 nm
